# Clinicopathologic features of 112 cases with mantle cell lymphoma

**DOI:** 10.7497/j.issn.2095-3941.2015.0007

**Published:** 2015-03

**Authors:** Dong-Mei Zhou, Gang Chen, Xiong-Wei Zheng, Wei-Feng Zhu, Bao-Zhen Chen

**Affiliations:** Department of Pathology, Key Laboratory of Cancer Translational Medicine of Fujian, Teaching Hospital of Fujian Medical University, Fujian Tumor Hospital, Fuzhou 350014, China

**Keywords:** Mantle cell lymphoma (MCL), immunohistochemistry, fluorescence in situ hybridization (FISH), prognosis

## Abstract

**Objective:**

This study aims to explore the clinicopathologic features of 112 patients with mantle cell lymphoma (MCL).

**Methods:**

Data from 112 MCL cases were collected, and immunohistochemical assay was conducted. Fluorescence in situ hybridization (FISH) detected a break in the CCND1 gene. The *t*-test was used in the statistical analysis.

**Results:**

All tumor cells in the 112 cases expressed B cell-related antigen, including 1 blastoid subtype and 1 polymorphic subtype. Among all cases, 106 expressed CD5 and 104 expressed cyclin D1. A break in the CCND1 gene was not found in 3 cases with CD5-MCL. IgH/CCND1 polyploid was observed in 2 classic cases.

**Conclusion:**

MCL is a type of special immunophenotypic B-cell lymphoma. The prognoses of blastoid and polymorphic subtypes are poor. Special subtypes should be classified during diagnosis.

## Introduction

Mantle cell lymphoma (MCL) is a rare type of non-Hodgkin’s lymphoma. It is strongly invasive, and its long-term survival rate is low. No standardized treatment has been established for this disease. This study analyzed clinical pathological data from 112 cases of MCL, in order to increase awareness, develop diagnostic techniques, and provide reference for clinical treatment and prognosis.

## Materials and methods

### Materials

#### Cases and specimens

Data from 112 cases of MCL were obtained from Fujian Provincial Tumor Hospital’s external examination and consultation conducted from January 2004 to May 2014. The follow-up time of these cases ended in May 2014. This study was approved by the ethics committee at Fujian Provincial Tumor Hospital University. The collected data were rechecked, and the cases were diagnosed by a combination of history, morphology, immunohistochemistry, and other relevant tests by two deputy director doctors. All specimens were prepared, and H&E staining and immunohistochemical staining were performed.

#### Antibodies used in immunohistochemistry

CD20 (MX003), CD79a (SP18), Pax5 (SP34), CD3 (polyclone), CD5 (SP15), CD21 (2G9), CD23 (1B12), CD10 (56C6), cyclin D1 (DCS-6), TdT (SEN28), and Ki-67 (MIB-1). These antibodies were purchased from Fuzhou Maixin Biotechnology Development Limited Company.

### Methods

#### Immunohistochemical staining

Paraffin sections were dewaxed in xylene, hydrated using graded ethanol, and then placed in the repair liquid of sodium citrate (0.01 mol/L, pH 6.0). Antigen retrieval was conducted in a pressure cooker. H_2_O_2_ solution [3% (volume fraction)] was added for 10 min to eliminate the activity of endogenous peroxidase. The sections were applied with drops of ready-to-use primary antibody, stored in a refrigerator for 4 °C overnight, applied with secondary antibody (PV9000 Immuno-Bridge; GBI Company, USA), and placed in a water bath at 37 °C for 30 min. The sections were then colored with freshly prepared diaminobenzidine. PBS was used instead of primary antibody as a negative control. Several organizations, which have positive antibody proteins, were employed as positive control.

Determination of results: The positive expression of substance was brown. The expression site of CD20, CD79a, CD5, CD21, CD23, and CD10 was the membrane and that of Pax5, cyclin Dl, TdT, and Ki-67 was the nucleus. Cell membrane and cytoplasm were the expression sites of CD3.

#### Fluorescence in situ hybridization (FISH) detection

IgH/CCND1 Probe was purchased from Vysis (USA). Hybridization step was carried out following the method provided in www.vysis.com (Vysis). Results were observed under a fluorescence microscope with reactive lymphoid tissue as normal control.

IgH probe of green fluorescence hybridization with two target fragments of IgH on 14q32 was marked. CCND1 probe of red fluorescence hybridization with approximately 350 kb fragment on 11q13 was marked. Two red signals and two green signals of t (11;14) (q13;q32) appeared in the nucleus and hybridized with target fragments on 11q13 and 14q32. No yellow fusion signal or green and red signals appeared close to each other in t (11;14) (q13;32). FISH was conducted to determine whether the cells of red and green separated signals in the tumor area, which were greater than 15%, were positive.

### Statistical analysis

SPSS 11.50 software was used for statistical analysis. The *t*-test was conducted, and *t* represents the differences between two sample averages. *P*<0.05 was considered statistically significant.

## Results

### Clinical features

Among the 112 cases of MCL, 91 were males and 21 were females. Their ages ranged from 27 to 78, with an average age of 58. Eighty-seven cases originated from the nodes; the most common symptom was extensive lymphadenopathy. Fourteen cases originated from the digestive tract, and they mostly manifested lymphoma polyposis. Eleven cases originated from the nasopharynx oropharynx, and 9 cases originated from the peripheral blood and bone marrow. The patients survived 3-90 months, and the average survival time was 37.6 months (**Table 1**).

**Table 1:** Comparison of Ki67 and survival of classic MCL with different immunophenotypes

Group	CD5^+^D1^+^	CD5^−^D1^+^	CD5^+^D1^−^	Total average
Cases	96	8	6	
Ki-67 (%)	44.1±0.072	42.5±0.097	42.5±0.086	44.8±0.064
Average survival time (months)	38.6±1.23	36.2±1.35	Lose contact	37.6±1.12

MCL, mantle cell lymphoma; D1, cyclin D1.

### Morphology

**Figure 1:**
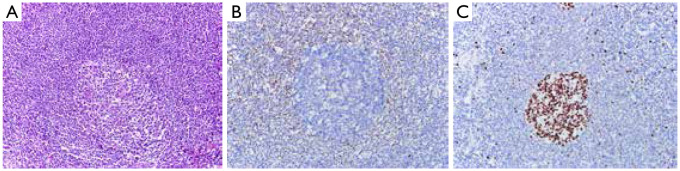
Mantle zone lymphoma. (A) The germinal center was surrounded by a thickened lymphocyte sleeve (H&E, ×200); (B) Immunohistochemistry: cyclin D1 (+) (Envision, ×400); (C) Ki-67 expression was low (Envision, ×400).

**Figure 2:**
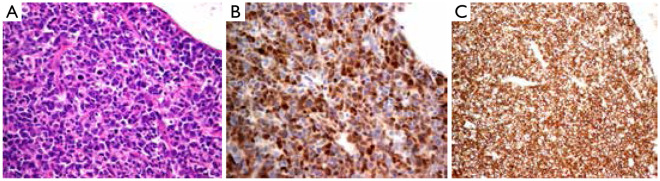
Polymorphic variant MCL. (A) Diffused tumor cells and polymorphic subtype with apparent nucleolus and high mitotic rate (H&E, ×400); (B) Immunohistochemistry: cyclin D1 (+) (Envision, ×400); (C) CD5 (+) (Envision, ×400). MCL, mantle cell lymphoma.

**Figure 3:**
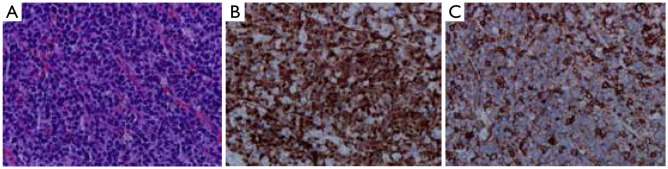
Blastoid variant MCL. (A) Blastic tumor cells (H&E, ×400); (B) Cyclin D1 (+) (Envision, ×400); (C) Immunohistochemistry: CD5 (+) (Envision, ×400). MCL, mantle cell lymphoma.

The 112 cases of MCL can be divided into mantle zone type (**Figure 1**), namely, nodular and diffuse, according to organizational structure. They can also be divided into classic and variant types (such as blastoid variant and polymorphic variant) according to cytologic morphology. The proliferation of single lymphoid cell was visible and formed by a majority of small- to medium-sized cells. The nucleus showed a slightly irregular contour and was similar to the center cell, whereas the nucleolus was not evident. The tumors lack transformed cells (centroblast) or immunoblast cells; no proliferation center was observed. Hyaline degeneration of small vessels, which scattered in the absence of phagocytosis of epithelioid histiocytes, could be observed in some cases. One case of pleomorphic variant of MCL’s tumor cells showed a medium or large size and was diffused, with pleomorphic nuclei. Nuclei were mostly rounding, but some were irregular, distorted, and recessed, with dispersed chromatin. Nucleoli were visible, and high nuclear division rate was observed (**Figure 2**). One case of blastoid variant of MCL’s tumor cells was similar to lymphoblasts. It exhibited a medium size, and its nuclei were round, with sparse chromatin. Nucleolus was not evident, and high nuclear division rate was observed (**Figure 3**).

### Immunohistochemistry

**Figure 4:**
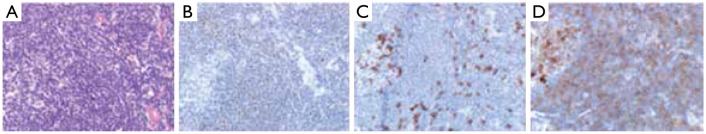
CD5^+^ CD10^+^ MCL. (A) Tumor cells showed a classic morphology, with the residual germinal center on the left (H&E, ×400); (B) Immunohistochemistry: cyclin D1 (+) (Envision, ×400); (C) CD5 (−) (Envision, ×400); (D) CD10 (+) (Envision, ×400). MCL, mantle cell lymphoma.

All of the 112 cases showed CD20, CD79a, and Pax5 positive, and CD3, CD10, CD21, CD23, and TdT negative. There were 106 cases with cyclin D1 positive, 6 cases with cyclin D1 negative, 104 cases with CD5 positive, 8 cases with CD5 negative, and 1 case with CD5 negative and CD10 positive (**Figure 4**). Ki-67 index was 5% to 90%.

### FISH results

**Figure 5:**
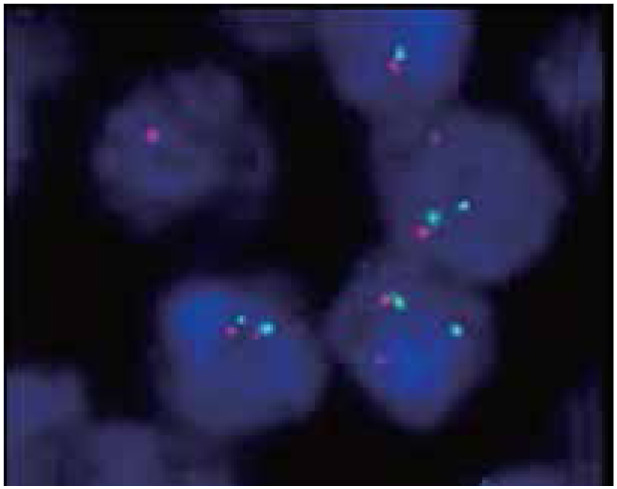
FISH image of MCL showing the CCND1 gene disruption (FISH, ×1,000); red/green signals marked the two ends of the CCND1 gene. A positive pattern showed two separate red and green signals. FISH, fluorescence in situ hybridization; MCL, mantle cell lymphoma.

**Figure 6:**
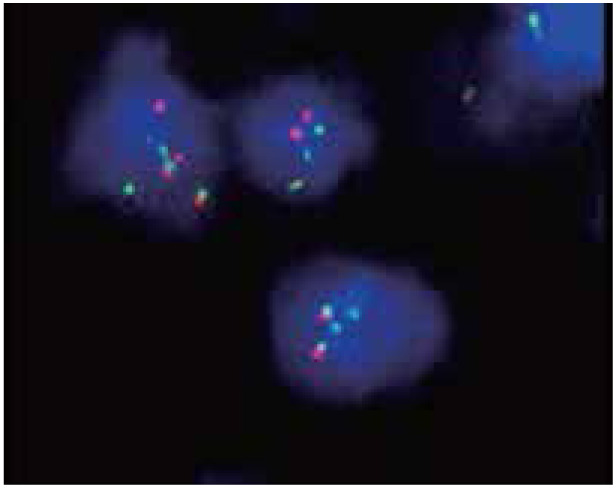
FISH image of MCL showing polyploidy (FISH, ×1,000). The diploid showed two red and two green signals, whereas the polyploid exhibited more than two signals. FISH, fluorescence in situ hybridization; MCL, mantle cell lymphoma.

The FISH results of 6 cases with negative cyclin D1 in MCL showed that the cell number of red and green separated signals was greater than 15%, indicating that chromosome translocation t (11;14) (q13;q32) existed. Among 8 cases of CD5 negative and cyclin D1 positive, 5 cases with the FISH results showed that the cell number of red and green separated signals was greater than 15%. This result implies that chromosome translocation t (11;14) (q13;q32) existed (**Figure 5**). The FISH results of 10 cases of CD5 positive and cyclin D1 positive in MCL showed that the cell number of red and green separated signals was greater than 15%, indicating the presence of chromosome translocation t (11;14) (q13;q32). Chromosome polyploidy appeared in 2 cases of them (**Figure 6**).

### Statistical results

Significant differences were not observed in Ki-67 expression and average survival time among groups of different immune phenotypes of classic MCL (group CD5^+^ cyclin D1^+^, CD5^−^ and cyclin D1^+^ and group CD5^+^ and cyclin D1^−^) (*P*>0.05). The cases of pleomorphic and blastoid variant groups were insufficient to make a comparison (**Table 1**).

## Discussion

MCL is a B-cell lymphoma. MCL was previously named intermediate lymphocytic lymphoma, centrocytic lymphoma, and diffuse small cleaved cell lymphoma. Given its unique clinical features, morphology, immunophenotype, and genetic characteristics, MCL is listed as an independent disease by the new WHO classification^1^. The incidence of non-Hodgkin’s lymphoma accounts for about 2%-10%^2^. This disease is more common in white than black people. It is also common in Asian population, with the average onset age of 60. Moreover, this disease is found more in males than in females. Lymph nodes are the most frequently involved sites. The extranodal sites involved include the spleen, bone marrow, gastrointestine, and Waldeyer ring. It is usually manifested as lymphoma polyposis in the gastrointestinal tract. In this group, the incidence rate was 2.8%, and the ratio of male to female was 6:1-7:1. The average onset age was 56, and lymph nodes were the most common involved site, which is similar to that reported in literature^3^.

### Differential diagnosis

At present, MCL is speculated to be derived from lymphocytes at the inner layer of the mantle zone without antigen stimulation. Its main morphological characteristic was the proliferation of single tumor cell. These cells were small to medium in size. Their nucleus was irregular and very similar to the center cell. Hyaline change in small blood vessels, which are scattered in epithelioid histiocytes (no phagocytosis), non-neoplastic transformation, and proliferation center were observed. The growth pattern can be divided into mantle zone, nodular, and diffuse types. It can also be divided into classic and variant (mother cell-like, pleomorphic, small cell, fringe-like, etc.) according to cell morphology. Immunohistochemistry showed the presence of B cells labeled CD19, CD20, CD22, and CD79a positive. In almost all cases, T cell related antigen CD5 was positive. CD43 was often expressed, but other T cell antigens were usually negative. Few CD23 was expressed, and CD10, BCL-6, MUM-1 were occasionally expressed and frequently appeared in variant MCL. These observations make the diagnosis difficult, particularly when the morphology is not typical or the sample is not enough.

Based on the aforementioned characteristics, tumors can be differentiated as follows:

- B small lymphocytic lymphoma (SLL). MCL small cell variant has round nucleus. It is composed of small lymphocytes and is similar to SLL. The proliferation center of SLL is similar to nodular MCL. SLL cells are composed of anterior lymph or immune cells. MCL cells are similar to the center cells. Their nuclei are slightly irregular, and transformed mother cell is rarely observed. When MCL CD5 and CD23 positive and cyclin D1 negative are found, SLL should be considered. When necessary, t (11;14) translocation can be detected.
- Follicular lymphoma (FL). FL nodular MCL exhibits a nodular growth pattern, which can be confused with FL. When single cell group, loss of central mother cell, and slightly irregular nuclei are observed, the possibility of MCL diagnosis is increased. However, if residual germinal centers in MCL exist, the center oocytes make the MCL diagnosis difficult. In histology, identifying between diffuse growth pattern of FL and diffuse type of MCL is difficult. Some MCL cells are CD5 negative, and others are CD10 and BCL6 positive. Thus, identification should be based on morphology and immunophenotype while rearranging gene is necessary.
- Marginal zone lymphoma (MALT). With marginal zone growth pattern, some MCL cells have extended to the marginal zone. MALT is derived from the outer layer of the mantle zone. The MCL cell is composed of various single nuclear cells, with plasmacytoid and interspersed transformed B cells. By contrast, MCL is derived from the inner layer of the mantle zone. No neoplastic transformed cells exist. Cyclin D1 staining showed that they are composed of tumor cells with little plasma.
- Lymphoblastic lymphoma. In blastoid subtypes, MCL cells are CD5, and cyclin D1 positive, and TdT negative, lymphoblastoid cells CD20, and cyclin D1 negative, and TdT positive.
- CD5-positive DLBCL. Pleomorphic subtype MCL cells are composed of large, heterogeneous cells. Nuclear fission is observed, and chromatin is exquisite. The inconsistencies between large nucleus and relatively small nucleolus may be attributed to the source of mantle cell. Cyclin D1 is essential to confirm diagnosis.
- Burkitt lymphoma. When blastoid subtypes appear, CD5 negative and abnormal expression of CD10 and BCL6 are identified. Cells should also be identified with Burkitt’s lymphoma because cyclin D1 is also expressed in this disease.

### Abnormal expression of MCL

MCL is not difficult to diagnose when typical morphology and typical immunophenotype are combined, but the group mark of some CD5 and cyclin D1 in MCL can be negative. Some studies^4,5^ shown that the expression of cyclin D1 in MCL were 50% to 70%, and 10% MCL with CD5 negative, and a few of MCL with CD10 positive. These phenomena are observed in some cases in this paper. Cyclin D1 negative may be related to tissue fixation, embedding, antibody sensitivity and specificity, and so on.

Some studies found that some cyclin D1 negative and their t (11;14) translocation can be detected, or cyclin D2 and D3 are expressed. However, the expression of the latter is not unique because they can also be expressed in other lymphomas. Thus, morphology should be considered or other tests should be performed for some cyclin D1 negative to avoid missed diagnosis and misdiagnosis. In this study, 6 cases in cyclin D1 were negative, as detected by FISH, and their t (11;14) translocation was detected and diagnosed. If the diagnosis was solely based on morphology and combination with other tests was not conducted, MCL should only be considered in the diagnosis. In addition, identifying pleomorphic MCL of cyclin D1 negative and DLBCL of CD5 positive is difficult. Therefore, a more specific antibody labeling is necessary. Recent studies have found that the transcription factor SOX11, a family member of SOX, can affect the Rb-E2F pathway and then regulates the expression of cyclin D1. SOX11 regulates the expression in MCL with cyclin D1 negative or positive^6,7^. It is obviously not expressed in mature lymphocytes; thus, it can be used as a specific biomarker of MCL. According to a recent report^8^, t (11;14) translocation was detected in a case of DLBCL of cyclin D1 positive, but its morphology and phenotype are typically DLBCL. While in another case, t (4;11;14) translocation was detected, but the morphology and phenotype are “gray zone lymphoma” between DLBCL and MCL. Therefore when the morphology and phenotype both support DLBCL, although (11;14) translocation is detected, the diagnosis of DLBCL of cyclin D1 positive cannot be excluded. In this study, there were 3 cases of CCND1/IGH negative detected by FISH among the 8 CD5^−^D1^+^ cases. A typical DLBCL morphology was observed in 1 cases. The morphology of the other 2 cases was between DLBCL and MCL. Thus, further tests are necessary to diagnose whether it is DLBCL or “gray zone lymphoma”.

### Prognosis

The long-term survival rate of MCL is lower than all lymphoma; its average survival period is 3 to 5 years^9^. Most MCL are incurable, and no specific effective treatment and specific prognostic indicators are available. Previous studies confirmed that the acquired gene conversion, as well as clinical progress, and shorter survival prognosis are closely related. MCL has four stages^4^. In the first stage, B cells obtain t (11;14) translocation, which is followed by a long incubation period. The second stage is an unknown mechanism; the cells begin to form *in situ* MCL or inert leukemia-like MCL by clonal proliferation. In the third stage, some cell parts are distorted. Important target groups are gained or lost, and then major cellular pathways are reduced. This stage enables cells to develop into a more malignant clone and form the classic MCL with invasive characteristics. In the fourth stage, malignant clonal cells undergo more genetic changes, such as deletion of 8q24, 9p21, and 17p13, activation of myc, and gene inactivation of CDKN2A and TP53. These changes may result in higher levels of cell proliferation, rapid disease progression, as well as changes in pleomorphic cells and blastoid cells. Consequently, the prognosis of mantle zone type (also known as *in-situ* MCL) is better than that of mother cell-like and pleomorphic variant subtypes. A literature reports that the average survival period of pleomorphic and mother cell-like variants is 3.8-17 months^10^, which is far less than classic MCL. Its mitotic rate is higher, and Ki-67 proliferation index is also higher. The Ki-67 proliferation index is the most important prognostic parameter of MCL in most studies. Previous studies also demonstrated that MCL mother cell-like or chromosome variant prone to polyploid and pleomorphic variant subtypes are related to high expression of cyclin D1^11^. Another study suggests that the prognosis of cyclin D1 negative is better^9^. In this paper, the Ki-67 index of the 2 cases of pleomorphic and blastoid variants was up to 90%, and the mitotic index ranged from 10 to 30/15 HPF. The disease rapidly progressed. Death within 3-6 months also shows that the prognoses of blastoid and polymorphic subtypes are poor. This study found the following interesting phenomenon: two CD5 and cyclin D1 positive in chromosome polyploidy were detected by FISH from the MCL cases during the CCND1 separation process. The two cases were diagnosed as late stage and involved the bone marrow. One patient died 46 d after diagnosis, and another patient is still under follow-up for 7 months after diagnosis. Confirmation is still needed whether a direct relationship exists between the polyploid in two cases and the progress in the late stage, and poor prognosis.

## Summary

In summary, subdividing the subtype is necessary for MCL diagnosis to provide reference for the clinical treatment and prognosis. MCL is a type of special immunophenotypic B-cell lymphoma, which has distinct clinical presentation, morphology, and genetic phenotype. Its occurrence and developed mechanism need more in-depth study of molecular biology. Results are expected to provide targets for specific treatment and provide a valuable biomarker for prognosis.
